# Health Problems and the Use of Medications and Traditional Therapies among Chinese Immigrants Living in Spain

**DOI:** 10.3390/healthcare9121706

**Published:** 2021-12-08

**Authors:** Bárbara Badanta, Aura Yolima Rodríguez-Burbano, Angeles C. López-Tarrida, Juan Vega-Escaño, Giancarlo Lucchetti, Sergio Barrientos-Trigo, Rocío de Diego-Cordero

**Affiliations:** 1 Research Group PAIDI-CTS 1050 Complex Care, Chronicity and Health Outcomes, Department of Nursing, Faculty of Nursing, Physiotherapy and Podiatry, University of Seville, 41009 Seville, Spain; bbadanta@us.es (B.B.); sbarrientos@us.es (S.B.-T.); 2 Program of Law, Faculty of Social, Political and Humanities, Universidad de Santander, Bucaramanga 680003, Colombia; 3 Department of Critical Care and Emergency, Hospital Saint John of God Aljarafe, Hospitaller Order of Saint John of God, 41930 Seville, Spain; angelacarmen.lopez@sjd.es; 4 Department of Nursing, Faculty of Nursing, Physiotherapy and Podiatry, University of Seville, 41009 Seville, Spain; 5 School of Medicine, Universidade Federal de Juiz de Fora, Juiz de Fora 36036-900, Brazil; g.lucchetti@yahoo.com.br; 6 Research Group PAIDI-CTS 969 Innovation in HealthCare and Social Determinants of Health, Department of Nursing, Faculty of Nursing, Physiotherapy and Podiatry, University of Seville, 41009 Seville, Spain; rdediego2@us.es

**Keywords:** health, drug therapy, traditional Chinese medicine, immigrant, Chinese

## Abstract

In this study, we investigate the health problems and the use of medications and traditional therapies among Chinese immigrants in the Southern region of Spain. A qualitative study using semi-structured interviews and including 133 immigrants and 7 stakeholders was conducted in 2017. Transcription, literal reading, and theoretical categorization were performed, and a narrative content analysis was carried out. The most common health problems were musculoskeletal (28.6%) and allergies (25.6%) related to work activity and unhealthy lifestyles. Key informants also reported gastric problems, stress, and changes in eating habits, mostly related to their work activity. For these problems, a large number of traditional remedies (herbs, diet therapy, acupuncture, vitamins, etc.) were used, usually combined with pharmaceutical drugs used for colds, flu, general malaise (29%), pain and fever (23%), and allergy drugs (9.2%). Chinese immigrants reported health conditions associated with their working conditions and life habits in Spain, using Traditional Chinese Medicine instead of pharmacological drugs. Understanding these health problems and promoting awareness towards traditional therapies in the healthcare system may help to design public policies and Health Promotion strategies targeting this group.

## 1. Introduction

There is a total of 258 million immigrants worldwide [1]. According to Eurostat data, 3.9 million people immigrated to one of the member states of the EU-27 in 2018, in which Spain ranked second (643,000) in the reception of immigrants in Europe [2]. Although the growing crises in sectors such as manufacturing, construction, and services derived from the global economic crisis that began in 2007 caused the departure of the foreign population living in Spain (such as people of Romanian or Moroccan nationality in the first half of 2017), other nationalities (e.g., Italians and Chinese) grew in absolute terms [3].

In general, newly arrived immigrants have better health than their counterparts in both the original and in the host country, and usually better health even when compared to the native population [4]. However, as time goes by, immigrants are usually exposed to worse living conditions and worse jobs than the native population [5], therefore a gradual deterioration occurs until reaching levels similar to those of the native population [6]. Immigrants also have worse self-perception of health than the native population [7,8].

Previous studies assessing the health of Chinese immigrant have already shown the prevalence of chronic diseases in this population. Although these are lower than in the native population (i.e., cerebrovascular diseases, chronic lower respiratory, cancer, etc.) [9], Chinese immigrants appear to have a higher prevalence of cardiac risk factors such as hypertension and diabetes after migrating to Western countries when compared with Chinese living in China, and the risk increases with a longer length of residence [10].

In the context of health problems, it is also important to understand the patterns of consumption of medications and its prevalence as compared to other traditional non-pharmacological remedies, which may have public health and clinical implications. A European study showed a significantly lower proportion of Chinese immigrants using pharmacological drugs as compared to natives and other immigrant groups [11]. The use of Traditional Chinese Medicine (TCM) is a way of expressing their cultural and ethnical identities [12]. They tend to use TCM rather than conventional medicine, while using the western system in emergencies [13]. Although traditional medicine is used to also address common pathologies in the native population, such as chronic pain or cancer [14], authors indicate the need of health staff to inquire about it. Health professionals usually do not ask about traditional therapies and, for this reason, they are commonly unaware of the interactions that sometimes arise from the combined use of both therapies [15].

Although Chinese is the fourth largest nationality of origin in Spain (4.6%) [16], clinical information systems have few data on this group. Therefore, there is an underrepresentation in studies related to health [17], due to language barriers, intense working conditions, and the use of traditional medicine [18]. The purpose of the study is to know the main health problems of Chinese immigrants living in Spain, to examine the use of pharmacological medications and traditional therapies among this Asian group and to discuss possible national policies on healthcare for the Chinese immigrants living in the South of Spain.

## 2. Materials and Methods

### 2.1. Design

A qualitative ethnographic design was adopted based on Roper and Shapira’s framework [19]. This approach allows us to explore a particular topic in a specific context and to perform an analysis focusing on subcultural groups rather than on entire societies. This approach is characterized by (a) a conceptual orientation provided by a research team; (b) focused on a specific community; (c) focus on a problem within a specific context; (d) a limited number of participants; (e) the use of participants who may hold specific knowledge; and (f) the use of selected episodes of participant observation [20]. Data collection consisted of semi-structured interviews and field notes, all conducted by the principal investigator over six months in 2017.

### 2.2. Setting and Sample

The study was conducted in Andalusia, the southernmost region of Spain and Europe, where, according to National Statistics Institute, 25% of the Chinese population of the region is concentrated.

Participants were recruited through Chinese businesses (e.g., bazaars, restaurants, grocery stores, fashion stores, technology stores, and wholesale businesses) and community institutions (e.g., educational institutions, Asian cultural centers, and health services) and, to increase the number of participants, a “snowball sampling” procedure was used.

Eligibility criteria included adult immigrants of Chinese origin who were able to communicate in fluent Mandarin Chinese, Spanish, or English; and migrated to Spain. A total of 252 businesses and institutions were visited and 133 Chinese immigrants agreed to participate. 

In order to include different perceptions and insights, we included a sample of participants with different profiles concerning age, jobs, and length of time living in Spain. The intention was to increase the credibility of our findings within the Andalusia areas and their transferability to other Spanish areas. Based on these inclusion criteria, our study sample included 133 participants. In addition, the Chinese community recommended relevant stakeholders during the first phase of the study, so they were selected based on their relationships, roles, and knowledge regarding the Chinese immigrant population. A total of seven interviews with stakeholders were also conducted (Table 1).

### 2.3. Procedure

Purposive sampling was used to select the participants [21]. They were identified and invited by the main researcher, who is a nurse with expertise in transcultural nursing and health inequalities. Field notes included information about witnessed events, verbatim verbal exchanges, and the researcher’s personal interpretations of events. Informal conversations and the interviews allowed the researcher to examine if interpretations of meanings behind observed behavior coincide with participants’ own understandings.

The goal of the semi-structured interviews was for participants to be able to expound on subjects they were concerned about, while also giving the researcher the opportunity to ask for elaborations about specific topics, explanations of observed events, and clarification of ambiguities. Interviews were carried out face-to-face, in Spanish, and lasted 20–30 min. The interviews happened in a place that the informants chose, such as their homes, workplaces, or at restaurants. They were audiotaped and transcribed verbatim by the main researcher and then, translated into English by a translation company. Data collection continued until data saturation was reached [22].

### 2.4. Instrument

The interview script was based on the idea that the main health problems of this population are related to living conditions and that the Chinese immigrants in Spain are not common users of the healthcare system, and nor do they use many western healthcare resources. The authors considered the National Health Survey of Spain (NHSS) [23], a nation-wide study collecting information on the population living in Spain, which included sociodemographic data, health status, and associated factors. The interview included a part of the population’s background, consisting of: demographic information (sex, age, marital status, level of education, length of residence in Spain, and employment status) and self-perception of health, which included some quantitative aspects, with questions addressing health status at the moment of the interview, health problems limiting activities of daily living, and work stress. We decided to use some closed questions to complement the qualitative interviews in order to provide quantitative information as well.

The interview script was sent to a group of experts in transcultural health (*n* = 3) that were responsible to evaluate the content of the script and assess its adequacy. All interviews were conducted using the following open questions: What do you think are the main health problems among Chinese immigrants, and what about you? Do different health problems appear when Chinese immigrants arrive in Spain? How do your ailments affect their daily life? Regarding the use of remedies and medications, what kind of elements do they usually use? On what occasions? Both parts of the interview are shown as Supplementary Material.

### 2.5. Data Analysis

The qualitative analysis was carried out following the steps proposed by Braun et al. [24]: (1) Familiarization with the data; (2) Generation of categories; (3–5) Search, review, and definition of themes; and (6) Final report (“P-number, sex, age/S-number”).

Data were captured through audio recording. Transcription, literal reading, and thematic categorization were performed. Data analysis started with individual readings to get an overview of the respondents’ experiences. Two researchers read all field notes and interview transcriptions several times, to gain an overall understanding of the content. The analysis continued by organizing descriptive labels, focusing on emerging or persistent concepts and similarities/differences in participants’ statements. The coded data from each participant were examined and compared with the data from all the other participants to develop categories of meanings. Two main themes were defined to reflect all of the categories: “Health problems” and “Health practices” (Table 2). In addition, quantitative analysis of some qualitative information was carried out using absolute numbers and percentages, as well as means and standard deviations.

### 2.6. Trustworthy

The consolidated criteria for reporting qualitative research (COREQ) were used to ensure that accurate and complete reporting of this study occurred [25]. The methods used for guaranteeing quality were data triangulation, including participants with different sociodemographic characteristics, and triangulation of data analysis via different researchers. Please see the completed checklist: ‘Consolidated criteria for reporting qualitative research’ (Supplementary Materials S1).

## 3. Results

The sample was composed predominantly of women (61.7%) with a mean age of 30.7 years and an average length of residence in Spain of 11.3 years. The majority of participants (78.3%) were from Zhejiang Province, China. The characteristics of the participants are shown in Table 3. After carrying out the qualitative analysis of our data, we decided to report immigrants’ and stakeholders’ quotations together, since these quotations support and are in line with each other, as seen in the sections below.

### 3.1. Health Self-Perception

Most participants had a good self-perception of health (56.8%) and 35.6% perceived they had a fair health, with a mean score of health self-perception of 77.1 (*SD* = 15.7) (ranging from 0 to 100). Participants with the best health self-perception were those who had been living longer in Spain (*U* = 1642.5; *p* = 0.03). Those who perceived their health as being good have been living in Spain for an average of 12.2 years (*SD* = 5.9), compared to 10 years (*SD* = 5.2) for those who perceived their health as being regular or poor.

Employment is the main reason why Chinese people migrate to Spain, and they take it as a way of life. In fact, dedicating as much time as possible to work is a characteristic learned in China: (P-88, man, 35 years) “*Everyone says to work like a Chinese (…). People are already used to working hard (…) and not like in Spain, if you do not work the government helps you, but in China there is such a large population and if you do not work, nobody will help you*”. Therefore, the conceptualization of work has a positive connotation that equates to health and quality of life. As time goes by living in Spain, working conditions improve (they change from employees to business owners) and therefore, they associate it with better physical and mental well-being.

This cultural behavior may help explain why 71% of participants said that health problems did not limit their daily life and 96.2% have not had any illness or health problem that has limited their normal activity for 10 consecutive days. Limitations of habitual activities because of health problems were not serious (28.4%) and these limitations were mostly associated with physical factors (66.7%).

### 3.2. Health Problems

Work activity and the stress derived from it, exposure to allergens, changes in eating patterns, as well as other unhealthy lifestyles (i.e., sedentary lifestyle, alcohol use, and smoking), are the main causes attributed to health problems of the Chinese immigrant population in this study (Table 4).

Among these problems, musculoskeletal conditions stand out, since the daily work activity is carried out for years, with forced postures that increase the risk of bone pain and muscle contractures. Although they point out that working conditions are equal for women and men, it is observed during our visits, that the heaviest physical work is mainly done by men. Spending most of the time working makes it difficult to acquire healthy habits such as physical activity. Therefore, this has a negative impact on the health of Chinese immigrants, causing increased blood pressure and other chronic diseases. (P-104, woman, 26 years) “*In my generation, people prevent much (…), but the generation of my parents did not (…). Formerly as they did not visit much to the doctor, they did not tell me a thing, but now I’m noticing many people with chronic diseases like diabetes, hypertension, asthma*”.

It was also common to observe that Chinese immigrants did not eat in appropriate spaces (e.g., dining rooms); they ate very quickly and in little quantity in order to return to their tasks as soon as possible. They are the ones who explain these issues as the origin of their gastrointestinal problems.

Regarding psychological problems, the participants pointed out work stress. The average score of work stress was 4.2 (*SD* = 1.57) (ranging 1 to 7). The stress levels were higher in men (*M* = 4.73, *SD* = 1.53) as compared to women (*M* = 3.85, *SD* = 1.51), most likely justified by the fact that men usually emigrate first and, as reported in our interviews by them, the success or failure of their businesses were a family responsibility, and this burden seems to increase stress.

### 3.3. Health Practices

The information obtained from the interviews allowed the researchers to understand the different “levels of care” that are used by the Chinese immigrants to address their health problems. The first level of care in this community is the use of traditional medicine despite the government healthcare system. In the case of an unsuccessful treatment, participants search for the second level of care, which is provided by the health professionals of the official Spanish health system. According to the participants, they prefer to make use of traditional remedies because they have easy and fast access and this allows them to continue working hard in their businesses.

### 3.4. Traditional Chinese Medicine

During the interviews, immigrants showed the interviewer bottles of several traditional medicines with labels in Chinese, which demonstrates the confidence this population has in Traditional Chinese Medicine compared to Western medicine. Specifically, the use of TCM is common for health problems that are considered “less serious”. Chinese immigrants have a great knowledge of the use of traditional medicine, easy access and, in general, its use is faster than going to public health services. Both pills and herbal products can be purchased in Spain or brought from China:

(P-85, man, 37 years) “*Most Chinese always bring their medicines from China. For colds, muscle pain, contractures, diarrhea, general problems (…). Usually, they endure without going to the doctor, they self-medicate and that way usually the problem is cured*”.

There are people who do not use them regularly in Spain, but they do when they return to China because of family recommendations: (P-100, woman, 25 years) “*Many times when I was a child and my head ached, my mother gave them to me (…); we have ointments, creams*”. In this way, the TCM can be used exclusively or combined with the Western treatments:

(P-130 man, 53 years) “*My mother was on chemotherapy, but she was all the time taking a Chinese medicine since she was diagnosed (…). Every day she took one. She died practically in a month, but until that month she remained as before*”.

The ways to apply TCM are diverse. The techniques and remedies used are based on theories of restoring body balance and maintaining health through energy:

(S-1) “*They use a lot of TCM: acupuncture, suckers (bá guàn), plants (…) for internal issues that they think are more related to a possible energy imbalance…For surgery problems… Chinese medicine has little to do (…). They identify foods with elements of the TCM, classify them in different ways and use them to restore internal balance*”.

### 3.5. Pharmaceutical Drugs/Medications

Contrasting Chinese and Spanish health systems, Chinese immigrants report that the Western medicine tends to be curative and focused on the relief of symptoms, which differs from the preventive nature of TCM.

Regarding pharmaceutical drugs, Chinese immigrants tend to mostly use: colds/flu/general malaise drugs (29%), pain and fever medications (23%), allergy drugs (9.2%), antibiotics (7.6%), and antihypertensives (3.8%). Other drugs such as anxiolytics, sleep pills, statins, contraceptives, and hormone replacement therapy are less consumed, accounting for 3% or less. In the fieldwork, it was possible to note that participants with specific diseases were not taking the medication prescribed by the doctor.

A summary table of the narratives for each topic is shown in Table 5.

## 4. Discussion

The Chinese immigrants in our study discussed musculoskeletal, gastric, and allergy health problems that relate to their life and work in Spain and their sedentary lifestyle. However, these diseases seem to not severely limit their daily lives, as shown by their good self-perception of health. In order to treat their health conditions, immigrants reported a high usage of TCM, in detriment of pharmacological drugs.

One of the most remarkable aspects of the Chinese culture is the connection between working hard and having a good quality of life which, in turn, is also related to a better health [26]. According to participants, a wealthy condition is essential for a good health. However, the percentage of participants who reported having good/very good health (58%) is lower than in other studies with a Chinese population [27] and the native Spanish population (74%) in NHSS [23]. It seems that the first years in Spain are challenging and they prefer to handle their discomfort and continue working, aiming to have a better life in the future. This way of thinking may be associated with not seeing the doctor and having undiagnosed conditions. This situation is supported by previous studies, in which immigrants tend to be reluctant to manifest fragility and seek attention in health systems because they are afraid of losing their jobs [28].

In our study, Chinese immigrants have a higher prevalence of musculoskeletal disorders as compared to the European Health Survey in Spain (EHSS) for lumbar and cervical pain in the immigrant population (23%) [29,30]. This is most likely a result of the working conditions and excessive working load that immigrants are submitted to, and, in this context, health problems are commonly related to habits and lifestyles in this population [31].

Another frequent condition is duodenal or gastric ulcers, which were reported by 5.3% of the sample, exceeding 2% of the native population and 1.8% of foreigners [30]. These gastrointestinal problems are also supported by previous evidence [32], motivated by work stress, fast and irregular meals [26,33], inappropriate life habits (alcohol and tobacco consumption), and continued exposure to dietary elements such as lactose [34].

It is also interesting that allergies are common among the Chinese immigrants. The exposure to substances such as pollens or plants is common in Europe, but unusual in China. Allergies among the Chinese immigrants living in Spain exceed the numbers of both immigrant women (18%) and the native population (14%) in EHSS [30]. The appearance of respiratory problems is also a problem and the length of stay seems to be correlated to these problems. Previous authors have already documented this pattern, showing that non-US-born adults (among them Chinese immigrant) with 10 or more years of residency in the United States had higher odds of current asthma than those who arrived more recently [35].

An important finding of this study was the high use of TCM among Chinese immigrants. In accordance with previous studies, racial minorities tend to report a greater skepticism towards pharmaceutical drugs and use prescription drugs as a last resort [36]. Both our study and others [27] detected a greater use of traditional medicine, a greater use of self-medication, and a lower use of pharmaceutical drugs in Chinese immigrants as compared to the native population [6]. Participants have reported the efficacy of TCM in chronic conditions, which corroborates with a previous systematic review [37], preferring methods of manipulation with a naturalistic approach, considering them less invasive and with few adverse reactions.

Nevertheless, for acute conditions, immigrants tend to recognize that pharmaceutical drugs could relieve their symptoms faster (for colds, flu, fever, etc.) and they often combined these drugs with traditional remedies. Techniques such as suction or cupping are used as analgesics, anti-inflammatories, or tissue regenerators in order to treat rheumatism, joint pain, sprains, facial paralysis, asthma, lumbago, and conditions where it is necessary to “extract wind” [38]. They also use edible floral extracts [39] and they adopt a diet with restriction of cold and hot foods for certain health problems [33].

Finally, although the TCM is very familiar among Chinese immigrants, factors such as age and acculturation influence its use. TCM is more commonly used by older people of Chinese origin [32], and their use decreases with a higher level of acculturation [37]. In addition to older age, women and people with pathologies who do not receive medical assistance are those who most often use TCM, factors that should be taken into account by health staff [40]. These findings are important to health managers in order to develop preventive strategies.

### Limitations

The present study has limitations that should be considered when interpreting our findings. First, this is a non-representative sample and generalizability must be made with caution. However, to our knowledge, this study had a great sample size of Chinese immigrants in Spain. Second, the sample consisted predominantly of women, which could be justified by the fact that women were more open to the interviews and were more available in the visited places. Third, our data relied on the information provided by immigrants and not by medical charts or physicians’ information.

Despite these limitations, our study has important public health implications. Our findings revealed a great use of TCM among this population. Health professionals should be aware and question their Chinese immigrants about the use of such therapies and about a possible substitution of conventional pharmaceutical drugs in detriment of TCM. It is also important that training is available for medical staff regarding the use of TCM and the possible beneficial and adverse effects of this treatment. In addition, based on the percentage of musculoskeletal problems, more occupational and ergonomic interventions should be provided by governmental institutions in order to avoid these health problems. Future studies should further understand the routine and health habits of Chinese immigrants all over the world, comparing their opinions and attitudes with individuals living in China and describing the barriers of healthcare access among this population. Likewise, trials using public health interventions should be designed to investigate what type of intervention should be used to promote better health outcomes and to increase Chinese immigrant’s trust towards the conventional health system, allowing them to treat their conditions.

## 5. Conclusions

Chinese immigrants reported health conditions associated with their working conditions and life habits in Spain, using TCM instead of pharmacological drugs. Understanding these health problems and promoting awareness towards traditional therapies in the healthcare system may help to design public policies and health promotion strategies targeting this group.

## Figures and Tables

**Table 1:** Profile of stakeholders.

Identification	Characteristics
S-1	Male; 38 years old; Spanish. University professor of Chinese. Has a doctorate. Expert in Chinese language and culture, traveling extensively to China. Has strong ties with the Chinese community as well as with his home country, Spain.
S-2	Male; 41 years old; born in China. Company manager; 19 years in Spain. Director of a Chinese language school in Seville. Collaborates with the health systems to adapt their services to the Chinese community.
S-3	Female; 26 years old; born in China. Doctor; 6 years in Spain. Works in a public hospital in Seville and knows the most common diseases that affect the Chinese community and how to manage and treat this community properly.
S-4	Male; 53 years old; Ethnic Chinese (second generation). Freelancer. From one of the first Chinese families to arrive in Seville. Knows the history of Chinese immigration in Seville and the changes over the years.
S-5	Male; 41 years old; Spanish. Economist and entrepreneur. Expert in Spanish-Chinese relations. Business manager of the Chinese population in Seville. A very relevant individual to the Chinese business community. Has several Chinese friends and travels frequently to China.
S-6	Male; 18 years old; Ethnic Chinese (second generation). Medical student. He was born in Spain, but his whole family is Chinese. He is the youngest informant to provide an overview of the relationship with the immigrant family, the acculturation aspects, the contrast between Western and Eastern cultures, and access to healthcare.
S-7	Female; 49 years old; born in China. Director of the Chinese Cultural Center and Chinese language teacher; 23 years in Spain. She is responsible for promoting cultural and social activities among the Chinese immigrant community in Seville, having full interaction with this population.

**Table 2:** Emergent themes and categories.

Theme	Category	Description
Health Problems	Health Problems	The health problems reflect signs, symptoms, or diagnosed diseases identified by the Chinese immigrant population. In addition, perceptions of the causes of health problems or certain factors that contribute to perpetuating poor health conditions are also incorporated.
	Attributed Related Factors	
Health Practices	Traditional Chinese Medicine	The use of traditional medicine includes all popular health practices and preventive, curative, or palliative practices derived from beliefs, customs, and ancestral knowledge, not associated with the usual practices of care of Westernized health systems.
	Western health system	Chinese immigrants have a perception about the health problems that can be treated in each of the health systems. The use of one or the other is based on experiences, worldview of health shared with the eastern or western system, as well as perceived weaknesses of them.

**Table 3:** Participants’ characteristics.

Variable	Male *M* (*SD*)	Female *M* (*SD*)	Total *M* (*SD*)	Statistics *p* Value
Age (years)	33.1 (7.2)	29.2 (7.4)	30.7 (7.6)	*U* = 1457.5
				*p* = 0.003
Years residing in Spain	12.7 (5.7)	10.4 (5.5)	11.3 (5.7)	*U* = 4774.5
				*p* = 0.017
	***n* (%)**	***n* (%)**	***n* (%)**	**Statistics** ***p* Value**
Sex	51 (38.3)	82 (61.7)	133 (100)	-
Marital status				
Single	11 (21.5)	34 (41.5)	45 (33.8)	χ^2^ = 7.35 *p* = 0.062
Married	36 (70.6)	46 (56.1)	82 (61.7)	
Living with a partner (not married)	3 (5.9)	2 (2.4)	5 (3.8)	
Divorced	1 (2.0)	0 (0.0)	1 (0.8)	
Level of education				
Secondary or lower	41 (80.4)	62 (75.6)	103 (77.4)	χ^2^ = 0.41
Vocational or university	10 (19.6)	20 (24.4)	30 (22.6)	*p* = 0.521
Employment status				
Employed	50 (98.0)	78 (95.1)	128 (96.2)	χ^2^ = 0.74
Unemployed	1 (2.0)	4 (4.9)	5 (3.8)	*p* = 0.649

**Table 4:** Main health problems perceived.

Health Problems	Statements *n* (%)	Quoatations Related to Main Health Problems
Allergy, asthma, respiratory problems	35 (26.3)	(P-6, man, 39 years) “Almost everyone who has been here for more than three years has allergies”.
		(S-5) “Allergies (…) it is something that almost nobody has in China and when it arrives here suddenly has. The atmosphere here is better than in China, but they say it is due to the olive tree”.
		(P-38, man, 41 years) “Those who work in restaurants… being all day or many hours in the kitchen with poor ventilation can cause some risks for the respiratory tract”.
Cold, sore throat	32 (24.2)	
Chronic back pain (cervical or lumbar) and bone pain	60 (45.1)	(P-72, woman, 43 years) “Many hours of daily work without rest or closure during the half day and including full weekends (…) Although you continue working, the back is affected”.
		(P-5 man, 43 years) “Intense physical activity is what I do for 3 h, once a week when I unload the truck with the merchandise. In addition, all week I have moderate activity when I have to go shopping at the stores to replenish the daily basic merchandise. The most normal thing is that my bones and muscles ache”
Headaches/migraine	24 (18)	
Gastric problems and intestinal	20 (15)	(P-63 woman, 22 years) “Many people have gastropathy for changes in the food routine due to so many hours of work”.
		(P-40, man, 53 years) “You get tired of work at night and you put anything in your mouth”.
Fever	11 (8.3)	
Nervousness, depression, anxiety, difficulty sleeping	11 (8.3)	
Dental problems	10 (7.5)	
Skin problems	10 (7.5)	
Hypertension arterial, high cholesterol, and others heart’s illness	10 (7.5)	
Thyroid problems	5 (3.8)	
Arthrosis, arthritis, or rheumatism	4 (3)	
Constipation	4 (3)	
Bruises and wounds	3 (2.3)	
Dizziness	3 (2.3)	
Hemorrhoids	2 (1.5)	
Liver dysfunction	2 (1.5)	
Otitis	2 (1.5)	
Urinary problems	2 (1.5)	

**Table 5:** Summary table of the results.

Health self-perception	The perception of health improves over time in Spain. Chinese immigrants change from employees to business owners, and therefore they associate it with better physical and mental well-being.
	Despite suffering some symptoms or health problems, Chinese immigrants do not allow this to affect their daily (work) life.
Health Problems	Achieving success at work, forced postures and loads, as well as the alteration of schedules, generate stress, musculoskeletal, and gastrointestinal problems.
	In Spain, Chinese immigrants are exposed to allergens which are different from those in China, causing them allergies and other respiratory problems.
Health Practices	Chinese immigrants have easy access to TCM. It is used more frequently for minor ailments, and is more widely accepted by the entire Chinese community than Western medicine.
	Regarding medications, colds/flu/general malaise drugs, pain and fever medications, allergy drugs, antibiotics, and antihypertensives were the most used, due to their healing effects.

## Data Availability

The data presented in this study are available on request from the corresponding author.

## Supplementary Materials

The following are available online at https://www.mdpi.com/article/10.3390/healthcare9121706/s1, Table S1: Consolidated criteria for reporting qualitative studies (COREQ): 32-item checklist.
